# EBV Positive Diffuse Large B Cell Lymphoma with Negative Pan-B Cell Markers, Case Report, and Literature Review

**DOI:** 10.1155/2024/4803071

**Published:** 2024-07-18

**Authors:** Janna Shold, Lea Jukic, Daniel Farrell, Wei Cui, Da Zhang

**Affiliations:** ^1^ Department of Pathology and Laboratory Medicine University of Kansas Medical Center, 3901 Rainbow Boulevard, Kansas City, Kansas 66160, USA; ^2^ University of Zagreb School of Medicine, Zagreb, Croatia

## Abstract

Most B cell lymphomas are positive for one or more B cell markers including CD19, CD20, CD79a, or PAX5. However, rare cases of mature B cell lymphoma not expressing any B cell markers have been characterized and recognized as distinct diagnostic entities by current classification guidelines, including plasmablastic lymphoma, primary effusion lymphoma, and ALK-positive large B cell lymphoma. We present a case of pan-B cell marker negative, EBV positive diffuse large B cell lymphoma that is positive for OCT2, BOB1, and clonal immunoglobulin gene rearrangement that does not meet diagnostic criteria for any B cell lymphoma by current 4^th^ and 5^th^ Ed beta version WHO Hematolymphoid Tumors classification. In challenging cases like the one presented, utilizing OCT2 and BOB1 immunohistochemical stains can assist in determining B cell lineage. The WHO tumor classification system should consider adding OCT2 and BOB1 as alternative B cell lineage markers into their corresponding categories.

## 1. Introduction

Lineage of hematolymphoid tumors is usually determined by flow cytometry or immunohistochemical staining profiles performed on paraffin-embedded tissue. CD19, CD20, CD79a, and PAX5 are widely used as markers to determine B cell lineage from the pre-B cell to the plasma cell stage [1–4]. OCT2 is a transcription factor that regulates immunoglobulin gene transcription with its co-activator BOB1 has been shown to be both sensitive and specific in determining B cell lineage [1, 5].

B cell lymphomas that are nonetheless typically negative for traditional pan-B cell markers have been observed and well characterized, including ALK positive large B cell lymphoma, primary effusion lymphoma, plasmablastic lymphoma, HHV8+ large B cell lymphoma, and unclassifiable large B cell lymphoma [6–9], Table 1.

The current 5th Ed beta version WHO Hematolymphoid Tumors classification listed the following essential and desirable diagnostic criteria for EBV positive diffuse large B cell lymphoma [11]. Essential: (1) Partial or total architectural effacement of affected tissue. (2) Atypical lymphoid infiltrate composed of either sheets of large malignant cells or many scattered large, transformed cells of variable morphology including HRS-like cells in a richly cellular reactive background, often accompanied by necrosis. (3) Large cells confirmed to be of B cell lineage (e.g., CD20, PAX5, or CD79a expression). (4) EBV present in the majority of large B cells. (5) Absence of in-born or acquired immunodeficiency or a history of lymphoma. (6) Exclusion of other EBV-related lymphomas and lymphoproliferative disorders. Desirable: (1) EBV DNA detectable in serum or whole blood (in selected cases). We present a case of pan-B cell marker negative, EBV positive large cell lymphoma with a clonal immunoglobulin gene rearrangement and positivity for OCT2 and BOB1, which successfully determined B cell lineage.

## 2. Case Presentation

A 66-year-old male presented to his primary care physician with unintentional thirty-pound weight loss and fatigue. His laboratory results were significant for hemoglobin = 9.4 g/dL (reference range 13.5–16.5 g/dL), white blood cell count (WBC) = 3.81 × 10 ^ 3/*μ*L (reference range 4.5–11.0 K/*μ*L), hematocrit = 30% (reference range 40–50%), mean corpuscular volume (MCV) = 92 fL (reference range 80–100 fL), and platelet count = 75 × 10 ^ 3/*μ*L (reference range 15–400 K/*μ*L). A computed tomography (CT) scan revealed significant splenomegaly and diffuse soft tissue masses by the spleen and pancreatic tail. The patient was referred to hematology.

A more comprehensive evaluation and review of clinical records revealed thrombocytopenia beginning in 2020. Repeated laboratory results showed anemia (hemoglobin 9.1 g/dL), thrombocytopenia (82 × 10 ^ 3/*μ*L), and neutropenia (absolute neutrophil count 0.80 × 10 ^ 3/*μ*L; reference range 1.8–7.0 K/*μ*L), along with IgM (419 mg/dL; reference range 38–328 mg/dL) and IgG (2,476 mg/dL; reference range 762–1488 mg/dL) monoclonal gammopathies, and an increase in *β*2 microglobulin (11.9 mg/L; reference range 0.8–2.3 mg/L). These cumulative clinical findings of splenomegaly, worsening anemia, neutropenia, monoclonal gammopathy, and thrombocytopenia that persisted for three years raised suspicion for a slowly progressive B cell non-Hodgkin lymphoma.

The patient's clinical condition worsened, leading to hospitalization due to frequent falls. During this admission, bone marrow and inguinal lymph node biopsies were performed.

## 3. Materials and Methods

Formalin-fixed (10% neutral, buffered) paraffin embedded sections were stained by routine hematoxylin and eosin preparations. Immunohistochemical analysis was performed on Ventana automated machine using Dako antibodies. A panel of stains were performed, including markers for B cells (CD20, CD19, CD79a, and PAX5), T cells (CD2, CD3, CD4, CD5, CD7, CD8, and CD43), follicular dendritic cells (CD21), plasma cells (CD138), cytotoxic T cells (granzyme B and TIA1), hematopoietic precursors and Hodgkin lymphoma work up (CD45LCA, CD10, CD15, CD30, BCL2, and BCL6), cytokeratin marker (pan-CK), viral stains for HHSV8 and EBV, and proliferative index Ki67. EBV in situ hybridization stain is performed on a Ventana BenchMark Ultra using Ventana ISH iVIEW Blue Plus Detection Kit and EBER1 (Epstein–Barr Virus-Encoded RNA1) DNP probe. Additionally, OCT2 and BOB1 immunohistochemical stains were performed at Mayo Clinic reference laboratory. All immunohistochemical stains follow the manufacturer's protocol, and all stains were interpreted at the University of Kansas Medical Center. TCR gene rearrangement study was performed at Mayo Clinic reference laboratory. Mayo Clinic Laboratories use polymerase chain reaction (PCR) using a multiplex primer method based on the BIOMED-2 strategy to amplify the T cell receptor beta and T cell receptor gamma loci.

## 4. Results

**Figure 1:**
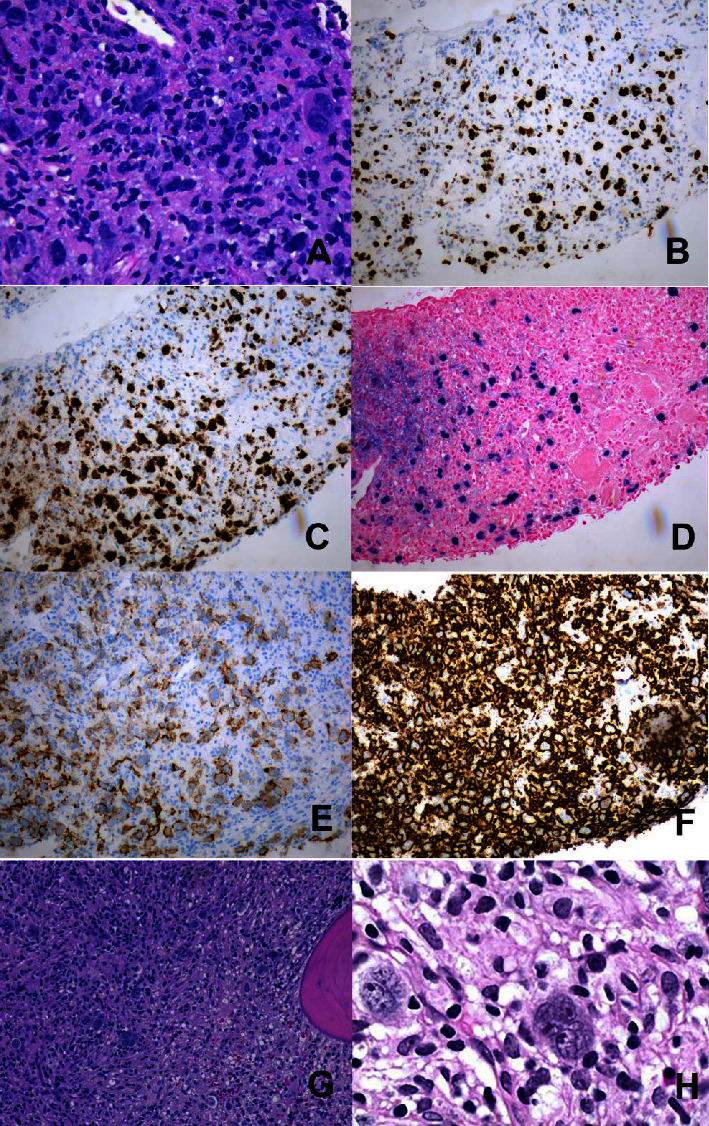
Representative sections of lymph node and bone marrow biopsies. The lymph node biopsy shows a diffuse infiltrate of large, atypical cells (A). The large, atypical cells were positive for Oct2 (B), BOB1 (C), EBV (D), CD30 (E), and CD45LCA (F). Representative section of the bone marrow core biopsy (G) and (H) shows infiltration of the marrow space by large, atypical cells.

Hematoxylin-eosin-stained sections of the lymph node showed a diffuse infiltrate of large, atypical cells in a background of small to medium lymphocytes, scattered histiocytes, and plasma cells (Figure 1(a)). The large, atypical cells were positive for CD2, CD30, CD45LCA, cMYC, BCL6, OCT2, BOB1, and EBV (Figures 1(b), 1(c), 1(d), 1(e), and 1(f)). They were negative for CD3, CD4, CD5, CD7, CD8, CD10, CD15, CD19, CD20, CD21, CD43, CD56, CD79a, CD138, ALK1, BCL2, fascin, pancytokeratin, PD1, PAX5, and HHSV8. Ki67 proliferation index was 40%. Background small and medium lymphocytes were predominantly T cells and B cells. B cell clonality testing detected a positive clonal immunoglobulin gene rearrangement. TCR gene rearrangement testing was equivocal.

The bone marrow biopsy showed a hypercellular bone marrow and involved by an atypical lymphoid proliferation consisting of large, atypical cells that were positive for CD2, CD30, CD45LCA, and EBV (Figures 1(g) and 1(h)).

The morphology and immunohistochemical staining pattern were consistent with a diagnosis of EBV positive diffuse large B cell lymphoma involving the bone marrow, and the patient started treatment with brentuximab vedotin, cyclophosphamide, doxorubicin, and prednisolone (BV-CHP). The case was also reviewed by Dr. Elaine Jaffe, NIH, USA, and the diagnosis was concurred.

## 5. Discussion

Pan-B cell marker negative B cell lymphomas are rare, most commonly observed in ALK positive large B cell lymphoma (ALK + LBCL), plasmablastic lymphoma (PBL), primary effusion lymphoma (PEL), HHV8+ large B cell lymphoma (HHV8+ LBCL), and unclassifiable large B cell lymphoma. The ALK + LBCL, PBL, PEL, and HHV8+ LBCL diagnoses can be made based on clinical presentation, morphology, ALK and HHV8 positivity, and expression of plasma cell associated antigens [1, 12, 13]. A small portion of cases of pan-B cell negative large B cell lymphoma, however, pose a diagnostic challenge and pitfall. Yin et al. [5] investigated the diagnostic utility of OCT2 and BOB1 for cases in which pan-B cell marker expression is negative. The cases included ALK + LBCL, PBL, PEL, HHV8 + LBCL, and unclassified large B cell lymphoma. Their study showed 74% of the cases were positive for OCT2, 85% of their cases were positive for BOB1, and 94% of their cases were positive for at least one of those markers. Importantly, non-B cell neoplasms were all negative for both OCT2 and BOB1. They concluded that OCT2 and BOB1 were both sensitive and specific for determination of B cell lineage. Chu et al. [12] investigated immunohistochemical markers to establish B cell lineage in CD20 negative B cell neoplasms. The cases included B cell lymphoblastic leukemia/lymphoblastic lymphoma, mature B cell neoplasms that were CD20 negative following rituximab therapy, and CD20 negative DLBCLs. For this discussion, the focus will be on the CD20 negative DLBCL cases. Their study noted that all cases were negative for CD79a and only one case was positive for PAX5. However, all the cases were positive for either OCT2 or BOB1. They concluded that OCT2 and BOB1 may be the most useful for determining B cell lineage in CD20 negative DLBCL cases, such as plasmablastic lymphoma and primary effusion lymphoma.

Similar to our case, Nakatsuaka et al. [14] presented a case of EBV positive diffuse large B cell lymphoma (DLBCL) that was negative for pan-B cell markers but positive for OCT2 and BOB1. Immunoglobulin gene rearrangement testing detected a clonal gene rearrangement. They concluded that utilizing OCT2 and BOB1 to determine B cell lineage in cases with unusual immunophenotype profiles can lead to the correct diagnosis and subsequent appropriate treatment.

**Figure 2:**
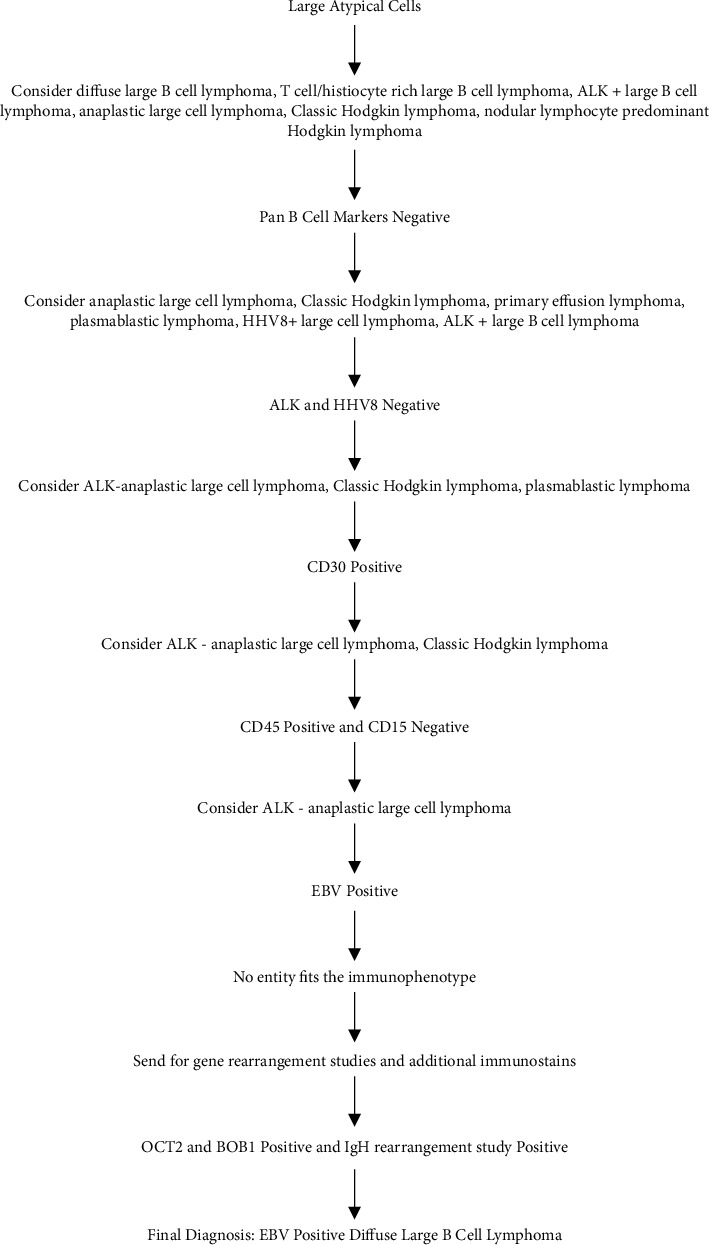
Decision tree for rendering the final diagnosis for this case.

Our case showed scattered, large, atypical cells in a background of lymphocytes, histiocytes, and plasma cells. The differential diagnosis initially included classic Hodgkin lymphoma, nodular lymphocyte predominant Hodgkin lymphoma, EBV positive DLBCL, T cell/histiocyte rich large B cell lymphoma, anaplastic lymphoma kinase (ALK) positive large B lymphoma, and anaplastic large cell lymphoma. The extensive immunohistochemical panel performed ruled out many of the possible diagnoses. The large, atypical cells were negative for pan-B cell markers, ALK, HHV8, and CD15, and positive for CD45LCA, which ruled out nodular lymphocyte predominant Hodgkin lymphoma, T cell/histiocyte rich large B cell lymphoma, classic Hodgkin lymphoma, ALK positive B cell lymphoma, and cavitary primary effusion lymphoma. While MUM1 was positive in our case, CD138 was negative, and the tumor cells did not show immunoblastic morphology, so plasmablastic lymphoma was less likely. The large, atypical cells were positive for CD2, CD30, Oct2, BOB1, and EBV and lacked the morphology of classic “Hallmark cells.” A clonal immunoglobulin gene rearrangement was detected, and TCR gene rearrangement was equivocal. The equivocal TCR result may indicate that the pattern of gene rearrangements is atypical compared to typical polyclonal T cell processes, but not definitive for a monoclonal T cell population. These findings led to the determination that this tumor was of B cell origin. A diagnosis of EBV positive large B cell lymphoma rather than ALK negative anaplastic large cell lymphoma or T cell lymphomas was rendered (Figure 2). Our case does not fit within a specific diagnostic category in the 4^th^ Ed or 5^th^ Ed beta version of WHO Classification of Tumors Hematolymphoid Tumors, and it is important to consider utilizing OCT2 and BOB1 to determine B cell lineage for lymphoma that is negative for pan-B cell markers. We believe these markers should be incorporated into future editions of the WHO Classification of Tumors Hematolymphoid Tumors.

## Figures and Tables

**Table 1:** Differential diagnosis for a lymphoma that is negative for pan-B cell markers.

Diagnosis	Essential criteria	CD30	ALK	EMA	CD138	HHV8	MUM1
ALK + LBCL	(1) Large cell morphology; (2) plasmablastic immunophenotype; and (3) ALK expression	−	+	+	+	−	+
PBL	(1) Lymphoma with plasmablastic/immunoblastic morphology; (2) expression of plasma cell-associated antigens; and (3) negativity for CD20, PAX5, ALK, and HHV8	−	−	−	+	−	+
PEL	(1) Presents as a serous effusion in the pleural, pericardial, or abdominal cavity or tumor mass associated with effusion; (2) absence of lymph node or other extranodal involvement; (3) large pleomorphic malignant cells with the immunophenotype of terminally differentiated B cells; and (4) positive for HHV8	+	−	+	+	+	+
HHV8 + LBCL	(1) Presents with primary nodal and/or splenic involvement; (2) effacement of architecture by large blasts/transformed B cells with generally plasmablastic, immunoblastic, or anaplastic morphology; and (3) positive for HHV8	−	−	−	−	+	+

The essential criteria are per the 5th edition of the World Health Organization classification of hematolymphoid tumors [10].

## Data Availability

Data present in this case report are available upon request.
